# Whole-genome resequencing and transcriptional profiling association analysis revealed the intraspecies difference response to oligosaccharides utilization in *Bifidobacterium animalis* subsp. *lactis*

**DOI:** 10.3389/fmicb.2024.1375384

**Published:** 2024-04-09

**Authors:** Zhenghui Lan, Xueling Zhang, Meng Xu, Junkai Kong, Xuancheng Zuo, Yixuan Wang, Chenxi Wang, Yingdi Teng, Yongqing Ni, Yan Zhang

**Affiliations:** School of Food Science and Technology, Shihezi University, Shihezi, Xinjiang Province, China

**Keywords:** *Bifidobacterium animalis* subsp. *lactis*, oligosaccharide metabolism, genotype comparison, resequencing, real-time PCR

## Abstract

**Introduction:**

As prebiotics, oligosaccharides are frequently combined with *Bifidobacterium* to develop synbiotic products. However, a highly diverse gene repertoire of *Bifidobacterium* is involved in sugar catabolism, and even phylogenetically close species may differ in their sugar utilization capabilities. To further explore the mechanism underlying the differences in *Bifidobacterium animalis* subsp. *lactis* oligosaccharide metabolism.

**Methods:**

This study screened strains with differential oligosaccharide metabolism. Subsequently, these strains were subjected to genome-wide resequencing and RT-qPCR.

**Results:**

The resequencing results indicated that the subspecies of *B. animalis* subsp. *lactis* had a high genome similarity. The RT-qPCR results revealed that glycosidase genes exhibited consistency in the phenotype of metabolism at the transcriptional level; the better the growth of the strains on the oligosaccharides, the higher was the expression of glycosidase genes related to the oligosaccharides. Our results suggested that the differences in the gene transcription levels led to intraspecies differences in the ability of the strains to metabolize oligosaccharides even when they belonged to the same subspecies.

**Discussion:**

Future studies with more sample size could generalizable the conclusion to all *B. animalis* subsp. *lactis* strains, thus would lay the theoretical foundation for the utilization of the *B. animalis* subsp. *lactis* strain as probiotics and the development of synbiotic products.

## 1 Introduction

The members of the genus *Bifidobacterium* are common inhabitants of the gastrointestinal tracts of humans and other mammals, where they ferment several diet-derived carbohydrates that cannot be digested by their hosts (O'connell et al., 2013). Until date, most studies have investigated *Bifidobacterium* and product development, such as the transformation of plant isoflavones by *Bifidobacterium*, the production of *Bifidobacterium*-fermented milk, and microencapsulation of *Bifidobacterium* (Gaya et al., 2017; Lohrasbi et al., 2020; Yan et al., 2020). The strains of *B. animalis* subsp. *lactis* are well-known health-promoting probiotics that are used commercially (Bunesova et al., 2017). The *B. animalis* subsp. *lactis* genome also contains a large number of transporters that transport complex carbohydrates, as well as genes for intracellular or extracellular hydrolases that degrade the glycosidic bonds in complex carbohydrates, which, in turn, facilitates the cell’s uptake of these complex carbon-hydrates and provides the bacterium with the energy required for growth, thereby adapting to a complex environment. The genome sequence of *B. animalis* subsp. *lactis Bl-04* reveals putative prebiotic transport and catabolic pathways, as reported elsewhere (Paineau et al., 2008), thereby implying that the bacterium is highly adapted to the gastrointestinal tract (GIT) and can utilize diet-derived complex oligosaccharides. Certain indigestible carbon sources (oligosaccharides) consumed daily stimulate the growth of *Bifidobacterium* in the gut, are commonly used as the main prebiotics (Hopkins et al., 1998). Changes in the dietary composition may affect health; therefore, it is important to study the ability of *Bifidobacterium* to utilize carbohydrates as a theoretical guide for the rational design of new symbiotic products (“prebiotic-probiotic”).

Oligosaccharides are carbohydrates formed by 2–10 monosaccharide molecules connected by glycosidic bonds. Owing to the rich variety of monosaccharide molecules that constitute oligosaccharides and the variety of intermolecular connection types and connection positions, there are several types of oligosaccharides in the nature. They can be broadly categorized into common oligosaccharides and functional oligosaccharides based on their biological functions. Common oligosaccharides mainly include lactose, sucrose, and maltose, which are easily digested and absorbed by the intestinal tract and thereby provide energy for the body’s metabolism, growth, and development (Vieira et al., 2020; Liu et al., 2023). Functional oligosaccharides, also known as non-digestible oligosaccharides, are difficult to digest and absorb because of the lack of an enzyme system in the human intestinal tract that directly degrades these functional oligosaccharides. The functional oligosaccharides developed so far mainly include xylo-oligosaccharides (XOS), galacto-oligosaccharides (GOS), fructo-oligosaccharides (FOS), inulin, and resistant starch (Tomás-Barberán and Mine, 2013; Belorkar and Gupta, 2016). Because functional oligosaccharides are not hydrolyzed by intestinal digestive enzymes but can be decomposed, absorbed, and utilized by beneficial microorganisms in the intestinal tract, they are often used in combination with Bifidobacteria to develop symbiotic products.

However, the main challenge of using *Bifidobacterium* cultures directly in the food is that different matrices and environments hinder their growth and survival in food (Moradi et al., 2021). To increase the survival rate of *Bifidobacterium*, combining probiotics and prebiotics (symbiotic) has become a research hotspot. Prebiotics are substrates that are selectively used by host microbes to confer a health benefit (Sanders et al., 2019). However, probiotic bacteria selectively ferment prebiotics comprising mainly non-digestible oligosaccharides (Abou et al., 2013). Therefore, research was needed to understand the factors and mechanisms that the *Bifidobacterium* exhibits intraspecies differences in oligosaccharide utilization The application of symbiotic products is limited by the fact that not all oligosaccharides are appropriate for all *Bifidobacterium*. The efficacy of specific combination warrants validation before use. A past study has shown that *Bifidobacterium* exhibits interspecies differences in oligosaccharide utilization (Garrido et al., 2015). For example, *B. longum* has been found to consume most oligosaccharides, and the *B. bifidum* strain utilizes GOS with degrees of polymerization (DP) of 2 and 3 and exhibits very limited use of FOS (Li et al., 2017). In addition, on examining three *Bifidobacterium* strains, the strains exhibited specificity in utilizing human milk oligosaccharides (LoCascio et al., 2007). The *B. longum* subsp. *infantis* are more inclined to have better capabilities to metabolize human milk oligosaccharides. The XOS, FOS, soya bean oligosaccharides, and malto-oligosaccharides are more effective in promoting the growth of *B. adolescentis.* However, surprisingly, the oligosaccharide utilization ability varies between different isolates of the same species (Garrido et al., 2015). The presence of intraspecies differences in *Bifidobacterium* creates further challenges when combining symbiotic products and using *Bifidobacterium*. Ang-Xin Song et al. recently reported strain-specific utilization of wheat arabinoxylan by *B. longum* (Song et al., 2020). Furthermore, a study demonstrated that several endogalactanase-positive (GalA^+^) *B. breve* strains can utilize purified GOS (PGOS) components with a high DP, whereas endogalactanase-negative (GalA^−^) strains cannot. This intraspecies difference has been ascribed to *galA* present in *B. breve* strains (O'Connell Motherway et al., 2013). *Bifidobacterium* strain specifically utilizes oligosaccharides; such discoveries have generated substantial interest in the scientific community. The characteristics of the strains must thus be considered while producing symbiotic products. While synthesizing symbiotic products, specific prebiotics must be designed to pair with specific probiotics that will consume them and exhibit improved functional outcomes. Therefore, revealing the mechanisms underlying *Bifidobacterium* intraspecies differences in the metabolism of oligosaccharides and explaining the regularity of these differences are highly warranted for the development of effective synbiotics.

Most of the current studies are based on genotype–phenotype to investigate interspecies differences in the metabolism of functional oligosaccharides by *Bifidobacterium* (Bottacini et al., 2018; He et al., 2021). In contrast, the genomic structures of *B. animalis* subsp. *lactis* strains are similar, therefore there is a lack of differences in the metabolism of functional oligosaccharides by *Bifidobacterium* that can be analyzed from the transcriptional level.

The present study focused on *B. animalis* subsp. *lactis* obtained from human fecal samples of two regions with significant dietary differences. Then, we investigated their strain specificity in terms of the oligosaccharide-utilization capacity at the subspecies level. Using genome resequencing technology explored the key loci associated with oligosaccharide metabolism, combined phenotypic and genotypic data, and identified gene sequences significantly associated with differences in the oligosaccharide metabolism. RT-qPCR was performed to determine the differential gene expression during the growth of *B. animalis* subsp. *lactis* on oligosaccharides. The purpose of this study was to explore differences in the utilization phenotypes of different carbon sources, and to associate possible gene clusters for revealing the strain metabolism difference of functional oligosaccharides in *B. animalis* at the subspecies level and provides a theoretical basis for the study of *B. animalis* subsp. *lactis* and the application of synbiotic products.

## 2 Materials and methods

### 2.1 Materials

FOS, GOS, XOS, isomalto-oligosacchrides (IMO), raffinose, stachyos, inulin, resistant starch (RS), and glucose (Glu) were all purchased from Shanghai Yuanye Bio-Technology Co., Ltd. All other chemicals used were of analytical grade.

### 2.2 Sample collection and treatment

In sterile containers, we collected the fecal samples of mothers and their infants residing in Hotan, Xinjiang, China and Hainan, China. The samples were collected at the participants’ homes and immediately frozen (−20°C) in a portable freezer. These samples were then transported to the laboratory and processed immediately upon receipt. The participants were not on any special diet, and, as instructed, had not taken probiotics or antibiotics for 2 months before sampling.

### 2.3 Isolation of *Bifidobacterium* from fecal samples by using the culture method

The samples were serially diluted (10^−1^ to 10^−4^) in an anaerobic chamber, and 0.1 mL of aliquot of the 10^−2^, 10^−3^, and 10^−4^ dilutions were plated on *Bifidobacterium* specific mMRS agar. The medium (per liter) was composed of yeast extract, 5 g; peptone, 10 g; beef paste, 10 g; Tween 80, 1 mL; sodium acetate anhydrous, 5 g; ammonium citrate dibasic, 2 g; K_2_HPO_4_, 2 g; MgSO_4_, 0.58 g; MnSO_4_, 0.25 g; glucose, 20 g; L-cysteine, 0.5 g (Blotopped, China); mupirocin, 0.05 g (Bomei, Hefei, China); nystatin, 0.025 g (Bomei, China), and powdered agar, 20 g (Ventura et al., 2006; Linares-Morales et al., 2022). All other reagents involved in the composition of the culture medium were purchased from Sinopharm Chemical Reagent Beijing Co., Ltd., China. These plates were subsequently incubated under an atmosphere of 10% H_2_, 10% CO_2_, and 80% N_2_ in an anaerobic chamber (DG520; DWS UK) and incubated for 48 h at 37°C (Shahin et al., 2003). All colonies appearing on the dilution plates were picked in succession and inoculated into mMRS broth.

### 2.4 DNA extraction and bacterial strain identification

The sample DNA was isolated from 1 mL of culture by using the FastPure Bacteria DNA Isolation Mini Kit (Vazyme, Nanjing, China) according to the manufacturer’s protocol. The purity and concentration of isolated DNA were measured using a micro nucleic acid quantitative instrument (Thermo Scientific, United States). DNA with a 260/280 nm ratio of >1.8 was used as the template DNA, and its integrity was determined using 1.2% agarose gels. Next, the strains were identified by conducting molecular biology assays, including 16S rRNA gene sequencing of genomic DNA and *Bifidobacterium* subspecies-specific PCR (*aptD* gene). PCR amplification of the 16S rRNA gene was performed with primers 27F (5′-AGAGTTTGATCCTGGCTCAG-3′) and 1492r (5′-CTACGGCTACCTTGTTACGA-3′) (Nomoto et al., 2017). To further identify the isolated strains, *atpD* was PCR amplified with primers atp-1 (5′-CACCCTCGAGGTCGAAC-3′) and atp-2 (5′-CTGCATCTTGTGCCACTTC-3′) (Ventura et al., 2004). The PCR-amplified products were detected through 1.2% agarose gel electrophoresis and sequenced using GENEWIZ (Suzhou Jinweizhi Biotechnology Co., Ltd., China). The sequencing results were submitted to the GenBank of the National Center for Biotechnology Information (NCBI) for BLAST homology comparison. A phylogenetic tree was constructed using MAGE 7.0 software. For repetitive-element PCR (rep-PCR), the strain was grown on MRS broth under anaerobic conditions for 48 h, and DNA was extracted as described earlier. Rep-PCR was conducted to generate genomic amplification products of the test strains. Specifically, rep-PCR amplification was performed using the Tc-512 PCR gene amplification instrument (Techne, British) and BOXAIR primers (Masco et al., 2003). The gel was visualized and photographed under a UV transilluminator (Quantum CX5, France). The separation of rep-patterns was processed with CelCompar ‖ version 6.6 (Applied Maths, Sint-Matenslatem, Belgium) and analyzed using the Pearson’s correlation coefficient and the unweighted pair group method with arithmetic mean (UPGMA) clustering algorithm.

### 2.5 Carbohydrate utilization

After 48 h of culture, the *Bifidobacterium* cells were centrifuged at 5000 xg for 5 min and washed twice with 0.9% normal saline. The cell suspensions (approximately 10^6^ CFU/mL) were used to prepare bacterial inoculants for the carbohydrate fermentation experiments.

The *in vitro* utilization experiment was conducted using oligosaccharides (FOS, GOS, XOS, IMO, raffinose, stachyos, inulin, RS) as the unique carbon source in a 96-well microplate (Kan et al., 2020). The carbohydrate solution with 1% final concentration was added to the medium, thereby replacing glucose as a carbon source. Four microliters of each resulting suspension were inoculated into 200 μL of the modified medium. The experiment was conducted thrice with triplicate wells (He et al., 2021). The medium in which glucose was added instead of oligosaccharides was used as the positive control, whereas the medium without any carbon source was used as the negative control. Moreover, 0.05% (w/v) L-cysteine was added to the medium to consume free oxygen and promote strain growth and reproduction. The cells were incubated at 37°C in an anaerobic chamber for 48 h. Each carbon source fermentation experiment was monitored by assessing OD at 600_nm_ by using a microplate reader (BioTek Instruments Inc., United States).

### 2.6 Statistical analysis of growth

The optical density (OD) obtained for each strain grown on different substrates was compared with that obtained for each strain in the absence of a sugar source. This difference in OD (ΔOD) was used as a parameter for evaluating each strain’s ability to grow on different substrates.

### 2.7 Whole-genome resequencing

The strains were cultured under anaerobic conditions in MRS broth containing 0.05% [v/w] L-cysteine hydrochloride at 37°C for 36 h. Chromosomal DNA was extracted from pure bacterial cultures by using the FastPure Bacteria DNA Isolation Mini kit. After DNA was extracted, DNA quantity and purity were assessed using the NanoDrop ND-2000 spectrophotometer (NanoDrop Technologies, Wilmington, DE, United States).

Then, 1 μg of genomic DNA was used to generate the sequencing libraries for each strain, followed by sequencing with the Illumina NovaSeq PE150 (Shanghai Biotree Biomedical Technology Co., Ltd.). The sequencing libraries were generated using the NEBNext® Ultra™ DNA Library Prep Kit for Illumina (NEB, USA) following the manufacturer’s recommendations, and the index codes were incorporated to attribute sequences to each sample. Briefly, the DNA sample was fragmented through sonication to achieve a size of 350 bp. The DNA fragments were then end-polished, A-tailed, and ligated with the full-length adaptor for Illumina sequencing, followed by PCR amplification. Finally, PCR products were purified (AMPure XP system). Libraries were analyzed for size distribution by using the Agilent 2,100 Bioanalyzer and quantified through real-time PCR.

After sequencing, sequence adapters, low-quality bases from paired reads, and reads with an average quality of <20 (<Q20) were first trimmed and filtered using fastp. Then, reads that passed quality-control filtering were aligned and assembled using SPAdes v3.15.3 with default k-mer sizes. Each assembly was annotated using prokka. The pairwise average nucleotide identity (ANI) values were calculated using pyANI v0.2.9 with default BLASTN+ settings (cut-off: ≥95% identity). Roary v.3.12.0 was used to obtain core genome data and perform multiple sequence alignment. The core genome phylogenetic tree was constructed using FastTree v2.1.9^1^ with the GTR model having 1,000 bootstrap iterations. Whole-genome sequence alignments for single nucleotide polymorphisms (SNPs) and indel identifications were performed using Snippy v3.13. Publicly available gene sequences of *B. animalis* type strains were downloaded from the NCBI Genome database. All *Bifidobacterium* genomes were input into dbCAN2 v2.0.1 [47] to annotate carbohydrate utilization-related genes based on the carbohydrate-active enzymes database (amino acid identity ≥30%, E-value ≤1 × 10^−5^). The *t*-test function implemented in SPSS v26 was used to calculate statistically significant differences between the average numbers of glycosyl hydrolase (GH) genes belonging to the predominant GH families (*p* < 0.05).

### 2.8 RNA preparation

Total RNA was isolated from 1 mL of *B. animalis* subsp. *lactis* cells (approximately 1 × 10^8^ CFU/mL) in the log-phase cultures grown on oligosaccharides or glucose as the sole carbohydrate source. The samples were centrifuged at 8,000 rpm for 1 min at 4°C on a bench centrifuge to collect cell pellets. The pellets were washed twice with 500 μL of DEPC-treated ddH_2_O to remove the culture medium and centrifuged at 10,000 rpm. To disrupt the collected cell pellets, they were ground in liquid nitrogen. The Spin Column Bacteria Total RNA Purification Kit (Sangon Biotech) was used for extracting the total RNA. The extracted RNA was eluted in DEPC-treated ddH_2_O. RNA degradation and contamination were monitored through 1.2% agarose gel electrophoresis. The electrophoretic results of the extracted RNA revealed that the bands of 23S and 16S rRNAs were bright and clear and that of 5S rRNA was darker. This finding indicated that the RNA extracted from the strains had good integrity, was less degraded, and could be used for the follow-up experiments. The RNA sample concentration was measured using the micro nucleic acid quantitative instrument to calculate the volume required for reverse transcription. The RNA samples were then immediately stored at −80°C until further use.

### 2.9 Real-time quantitative PCR analysis

Reverse transcription was conducted using the PerfectStart^®^ Uni RT&qPCR Kit (TransGen Biotech, China). The expression of oligosaccharide metabolism-related differential genes was detected through three RT-PCR replicates. Specific primers for each gene (Table 1) were designed using Primer Premier 6 software. The experiments were conducted using the Real-Time Q-PCR System (MX3000P, Stratagene, United States) in combination with the PerfectStart® Uni RT&qPCR Kit and the PerfectStart® Green qPCR SuperMix (TransGen Biotech, China).

**Table 1:** Target gene oligonucleotide primers for RT-qPCR.

Gene (Locus tag)	Primer sequence (5′ → 3′)	Product size (bp)
Balac_0475	GCTGACGATGGGAATGAC	160
	GCTCGACGTGTTCTACTC	
Balac_0483	CGTCGGAGTTCTTGATGG	142
	CAGGCAGCCTATGACTTC	
Balac_0484	CATGCCATGGGCTCAGCATCCACACAACATC	1750
	CTAGCTAGCTCAGCGCCTGAACGC	
Balat_0888	TGCCGTCGTGCTTCTGTT	876
	GCTCGTTGCGTGTGATAGG	
Balat_1241	CGCGGATCCATGACGATGACGTTCCCGAAGGGC	1,200
	CCGGAATTCCTACTTGGCGGAGTGCTCGGCGAT	
Balat_0977	CTTCGTTGTGCTTCTCGTTA	955
	CCATATTCGGATTGCGTGAT	
Balat_0373	GTCGCTTCATCAACTACACCTACATT	951
	ACACGCCATCCATCTGCTCAA	
Balac_1593	CTAGCTAGCGCTTCATGGTGGAAAAATGCTGTTG	1,632
	CCGCTCGAGTTCAATTACTTTGCTTATGAAAGCCTC	
Balac_1599	GAATTCCATATGGGCAGCGGGCAGGTCACGCTC	1,201
	CGCGGATCCCTACTTGCGGAAGTCACGAGCC	
Balat_0076	CGACGAATACGGCTACGACTG	578
	CGGCGAATGCGACCTTGTT	
Balac_0511	AACACCTCGTCGCTCTTCA	902
	TAGTTGTTGATGGTCGCCTTC	
Balac_0521	GCGATGCGATGCTGTGGAA	932
	ATTGGTGTTGCGTAGCGTCAT	
Balat_0517	CGCGGATCCAACCGGGCCGCCGTTTC	1,600
	CCGCTCGAGTTCACTCAATTCGCGGTAATC	
Balac_0514	GGCTGACCTTGGATTCTT	145
	CTTCTCGCCCATGTAGTTG	

The gene expression was normalized using the ∆∆C_T_ method, and groEL was used as the reference gene for the calculations. The control group consisted of strains cultured in MRS medium containing glucose as the carbon source, while the experimental group consisted of strains cultured in MRS medium containing each oligosaccharide as the carbon source. The experimental results were visualized by plotting heatmaps.

### 2.10 Statistical analysis

All experiments were three biological replicates. The data were statistically analyzed using SPSS V26 (IBM, Armonk, NY, United States). Data for all variables were normally distributed and allowed for parametric tests of significance. Equal variance data were analyzed by ANOVA, followed by Duncan’s multiple range test with 95% confidence intervals; the differences were considered significant at *p* < 0.05. Evolutionary trees were analyzed by MEGA7.0 software. A heatmap and the principal component analysis (PCA) were constructed in R software (ver 4.0.4).

## 3 Results

### 3.1 Isolation and identification of strains

**Figure 1:**
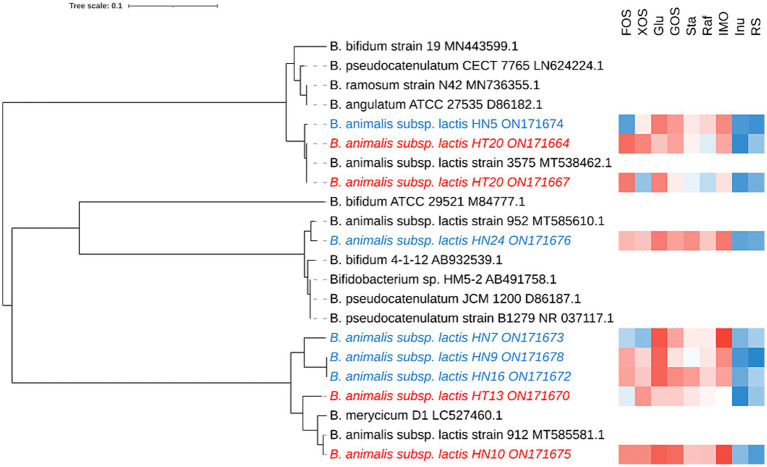
Phylogenetic tree and the functional oligosaccharide metabolism of *B. animalis* subsp. *lactis* strains. The color tending to red indicates that the strain grown better in the associated oligosaccharides, while that tending to blue indicates grown poorly in the associated oligosaccharides.

In total, 68 *Bifidobacterium* strains were obtained from different sources of fecal samples (Hotan Prefecture and Hainan Province) and 9 strains were screened through the rep-PCR and carbohydrate utilization, and the 16S rRNA gene sequence of 9 isolates was determined and compared with the published sequences obtained from the GenBank nucleotide database by using the BLAST algorithm. All 16S rRNA gene sequences reported in this study have been deposited at GenBank (Figure 1). Because our phylogenetic analysis based on 16S rRNA gene sequences revealed that all strains possessed high sequence similarities (99.5%) to *B. animalis* subsp*. lactis*, the isolates were tentatively identified as *B. animalis* subsp*. lactis.*

### 3.2 Growth of *Bifidobacterium animalis* subsp. *lactis* on different oligosaccharides

To investigate the intraspecies differences in oligosaccharide metabolism, we monitored the growth of *B. animalis* subsp. *lactis* strains on modified Rogosa medium containing 1% different oligosaccharides as the sole carbohydrate source. The results are shown in Figure 1, where strain-specificity exists in the utilization of sugars by different strains, but the general trend is that all strains exhibited vigorous growth on media containing FOS, XOS, Glu, GOS, raffinose, stachyose, and IMO as the sole carbon source. The higher growth performances of *B. animalis* subsp. *lactis* strains were noted on the media containing FOS, XOS, GOS, and IMO. HN5 exhibited poor growth on the medium containing FOS as the sole carbon source. HT20 and HN7 exhibited poor growth on the XOS-containing medium, whereas the other strains reached a high OD. When compared with FOS, XOS, GOS, and IMO, the strains grew moderately in raffinose and stachyose. Although most strains could utilize oligosaccharides, two exceptions were the utilization patterns of inulin and RS, where low or poor growth was observed in a few cases. HN5 and HN9 failed to grow in the media containing inulin and RS. All isolates from Hotan failed to grow in the inulin-containing medium. HN10 failed to grow in the RS-containing medium.

**Figure 2:**
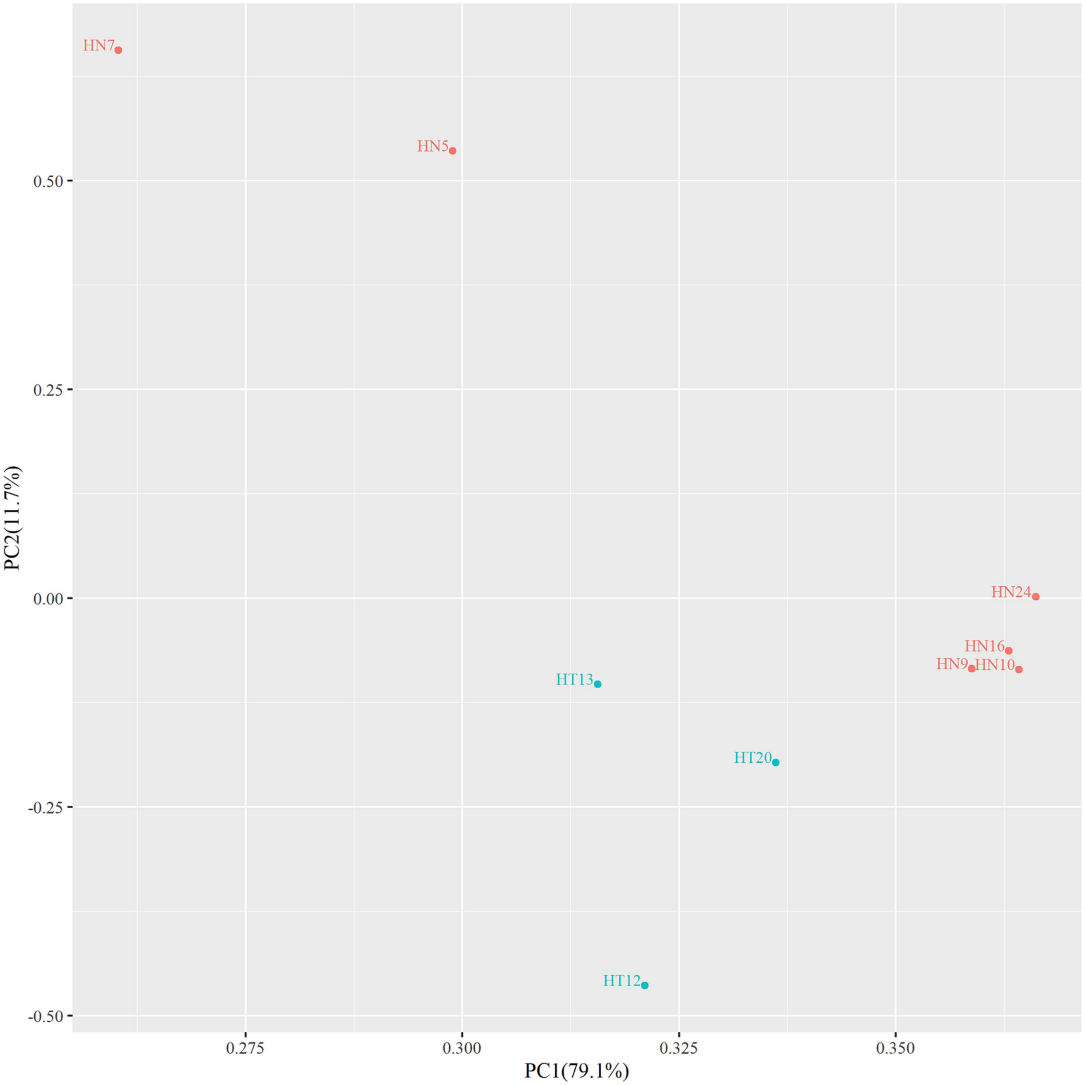
PCA diagram of functional oligosaccharide metabolism of resequenced strains.

In order to assess whether there were area differences in oligosaccharide metabolism among the resequenced strains, PCA analyzed experimental data on carbohydrate utilization. As depicted in the PCA diagram (Figure 2), the strains were clustered depending on the area. The results demonstrated that the selected strains from different areas exhibited differences in metabolizing functional oligosaccharides. The strains from Hainan (HN9, HN10, HN16, and HN24) and the strains from Hotan (HT12, HT13, and HT20) were clustered separately.

### 3.3 General features of *Bifidobacterium animalis* subsp. *lactis* isolates

To better comprehend the differences in the *B. animalis* subsp. *lactis* genomes, we resequenced 9 strains from China representing various geographical regions, including 3 strains from Hotan and 6 from Hainan. The results are shown in Table 2, and the genome size predicted for the strains ranged from 1.90 to 2.21 Mb. The genomes had an average G + C% content of 60.32%, an average predicted CDS number of 1,576, and the number of tRNA genes ranging from 53 to 59. In total, 1,452 core genes, 23 shell genes, and 171 cloud genes were identified in the *B. animalis* subsp. *lactis* genomes. The core genes, softcore genes, shell genes, and cloud genes were present in 99–100%, 95–99%, 15–95%, and 0–15% of the strains, respectively. The average nucleotide identity values were defined as the average base similarity between homologous segments of two microbial genomes. Nucleotide-level genomic similarities between the 9 genomes were determined using pyANI. The results indicated that *B. animalis* subsp. *lactis* ANI values ranged from 99.98 to 99.99% (Supplementary Table S1), indicating higher levels of sequence identity among all strains.

**Table 2:** Genome information for publicly available *B. animalis* subsp. *lactis* DSM 10140 and nine *B. animalis* subsp. *lactis* strains used in this study.

Strains	Ecological origin	Genome size (Mb)	No. Of CDS	No.Of tRNAs	GC (%)	Accession
HN10	Human feces	1.90	1,572	55	59.4	SAMN40528426
HN16	Human feces	2.03	1,574	54	60.38	SAMN40528427
HN24	Human feces	1.95	1,564	53	60.01	SAMN40528428
HN5	Human feces	2.03	1,572	55	59.65	SAMN40528429
HN7	Human feces	1.94	1,558	55	59.57	SAMN40528430
HN9	Human feces	2.15	1,596	59	60.69	SAMN40528431
HT12	Human feces	2.15	1,585	58	59.93	SAMN40528432
HT13	Human feces	1.95	1,560	54	59.74	SAMN40528433
HT20	Human feces	2.21	1,604	55	60.17	SAMN40528434
*B. animalis* subsp. *lactis* DSM 10140	Human feces	1.94	1,635	52	61.37	SAMN09742860

### 3.4 Glycobiome of the *Bifidobacterium animalis* subsp. *lactis* isolates

**Figure 3:**
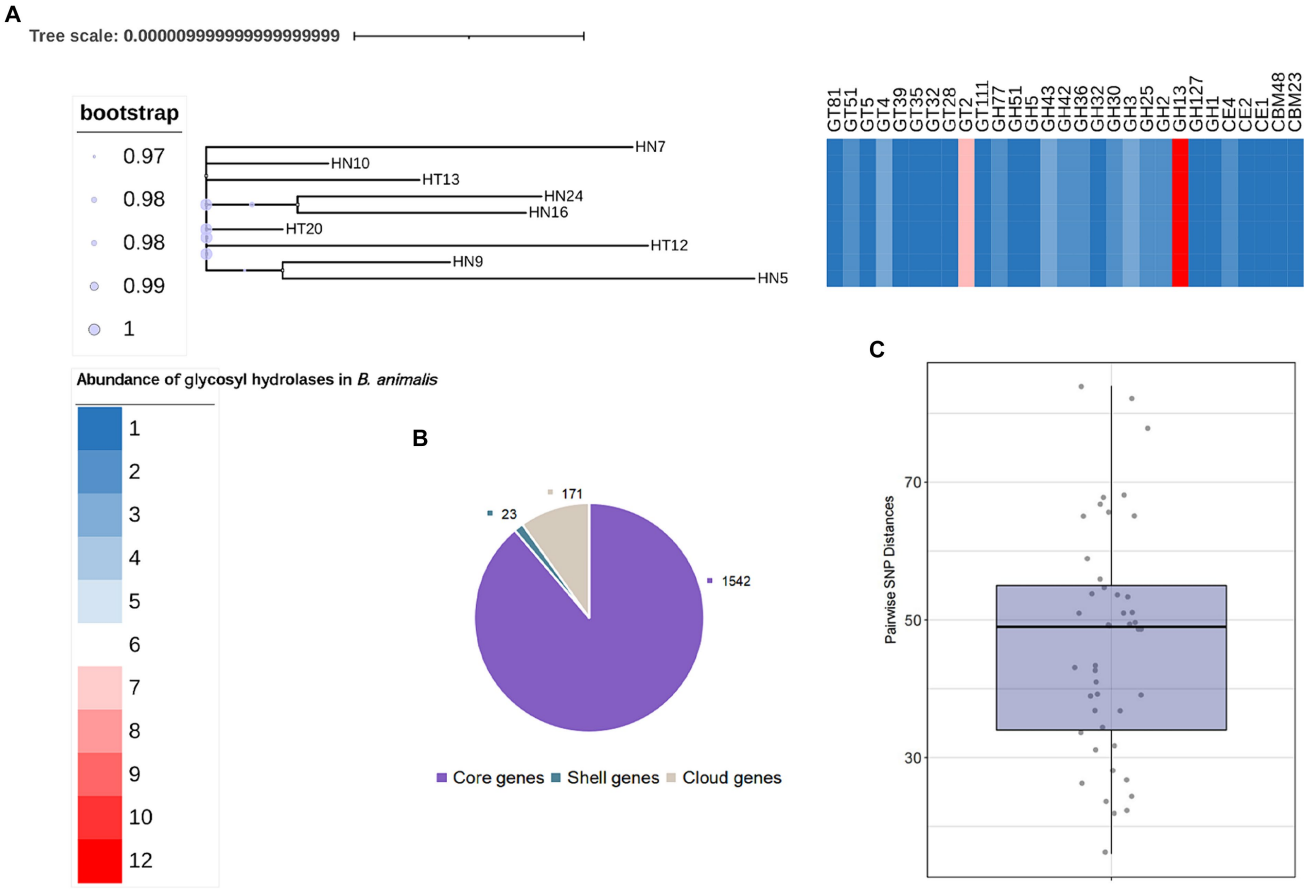
General Features of *B. animalis* subsp. *lactis* Genomes. **(A)** The maximum-likelihood trees were constructed based the core-genome alignment employing *‘*GTR*’*model with 1,000 bootstraps. Heatmap represents abundance of specific GH families. See also Supplementary Table S2. **(B)** Pan- and core genes of *B. animalis* subsp. *lactis*. **(C)** Pairwise SNP Distances between *B. animalis* subsp. *lactis*. Strains.

We further searched for the CAZymes profiles in *B. animalis* subsp. *lactis* genome. Of the 1736 gene families in the pangenome, 233 were determined to encode CAZymes, constituting 13.42% of functionally annotated genes, which reflects the saccharolytic character of the *B. animalis* subsp. *lactis.* These CAZymes included 315 glycosyl hydrolase (GH), 180 glycosyltransferases (GT), 36 carbohydrate esterases (CE), and 18 carbohydrate-binding motif-containing proteins (Figure 3). The most abundant CAZymes were GH13 (12 ± 0% GH genes per genome), followed by GT2 (8 ± 0% GH genes per genome), and GH43 and GH3 (3 ± 0% GH genes per genome). All these CAZymes encode enzymes associated with the hydrolysis plant-derived poly- and oligosaccharides, such as stachyose, raffinose and xylans, etc. And these poly- and oligosaccharides are major components of the adult diet. Studies have shown that the CAZymes profiles in *Bifidobacteria* genome are species and/or strain-specific, however, according to bioinformatic predictions, strain-specific CAZymes were not observed in this study (Genomics of the Genus *Bifidobacterium* Reveals Species-Specific Adaptation to the Glycan-Rich Gut Environment). On the contrary, regardless of the isolation source, all tested strains shared the same CAZyme repertoires.

We then explored the nucleotide-level differences in GH genes between the *B. animalis* subsp. *lactis* strains. The SNP analysis was conducted to compare the genomes of the 9 *B. animalis* subsp. *lactis* strains and *B. animalis* subsp*. lactis* DSM10140. Upon comparison of these 10 complete genome sequences, 493 validated SNPs were identified, and found that the *B. animalis* subsp. *lactis* strains exhibited low strain diversity, with the respective mean pairwise SNP distances of 46.73 ± 16.4 SNPs. Subsequently, these SNPs were functionally annotated. The results revealed that these SNPs were significantly associated with the transport and the metabolisms of carbohydrates (oligosaccharide). However, we found no significant SNPs that may cause functional changes in CAZyme genes. Interestingly, the experimental strains exhibited phenotypic differences in metabolizing different oligosaccharides. Therefore, we predicted that these SNPs were noncoding variants. Transcription factors play crucial roles in gene regulation by harboring these SNPs (Li et al., 2020).

### 3.5 Real-time quantitative PCR analysis

**Figure 4:**
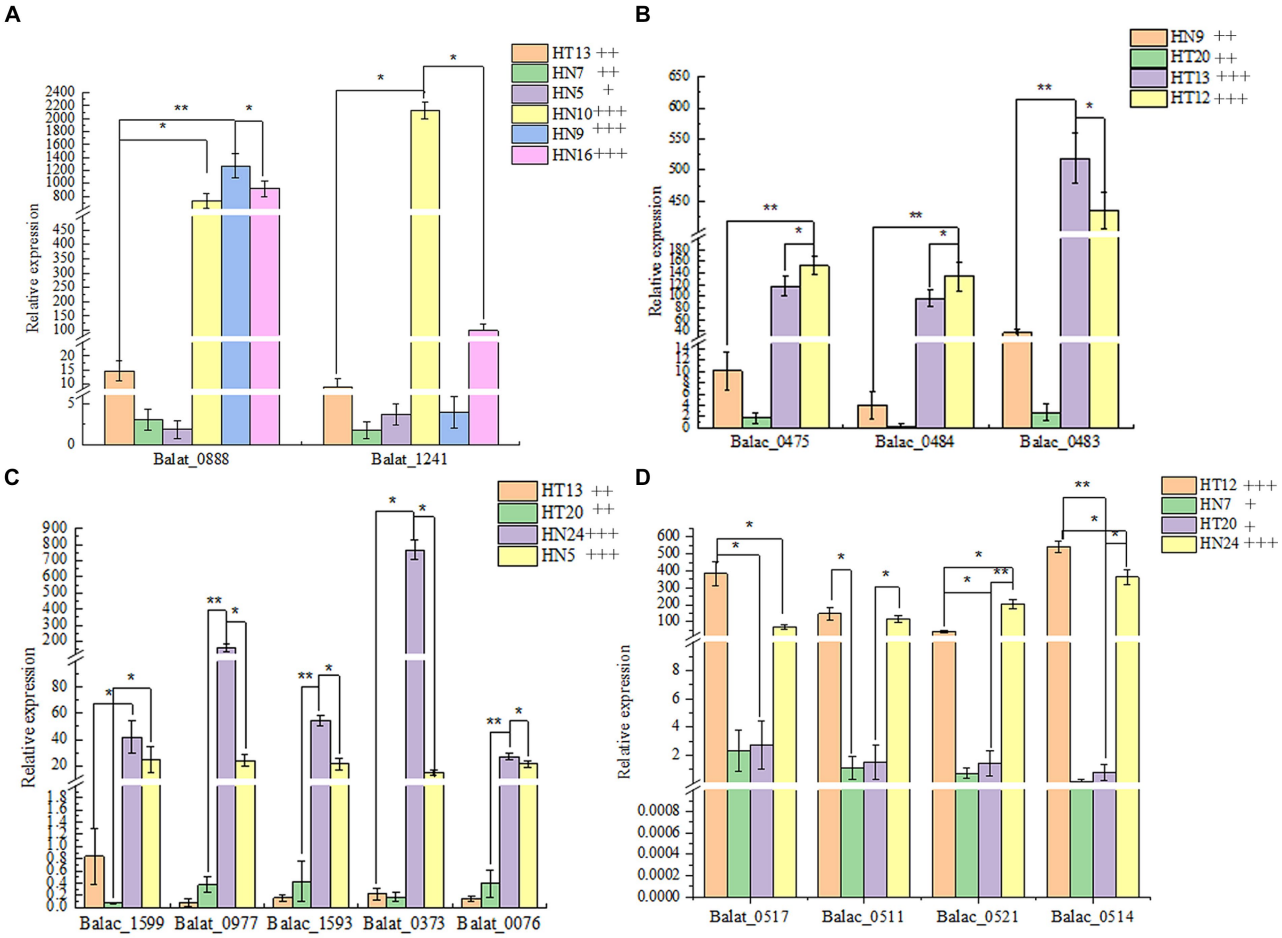
The relative expression of glycosidase genes in four oligosaccharides as the unique carbon source. **(A–D)**: Metabolic phenotypes of FOS, GOS, IMO and XOS and mRNA transcription levels of related genes by strains, respectively. The phenotype of metabolism was classified by the following labels: “+” OD600nm 0.25–0.5, low- level growth; “++” OD600nm 0.5–0.8, medium-level growth; “+++” OD600nm >0.8, high-level growth.

Thus, to reveal the phenotypic differences in the *B. animalis* subsp. *lactis* strains in metabolizing different oligosaccharides at the transcription level, total RNA isolated from the experimental strains was reverse-transcribed into cDNA, and then, the expression of relevant key enzyme genes was detected through real-time quantitative PCR (RT-qPCR). In the culture medium containing different functional oligosaccharides, the expression of the carbohydrate transporter gene changed to varying degrees at the mRNA level (Figure 4). The composition of oligosaccharides and monosaccharides are similar. However, their carbon chain lengths are different, and thus, they can induce the expression of the same or similar genes. On the other hand, a substrate can induce the expression of several carbohydrate metabolism genes. In this study, The results showed that the OD_600nm_ values of HN9, HN10, and HN16 strains were greater than 0.8 after 48 h of incubation in medium with FOS as the carbon source, and their relative expression of glycosidase genes (Balat_1241 and Balac_0888) related to FOS was higher than that of HN7, HT13, and HN5 (the OD_600nm_ values were 0.5 ± 0.04, 0.61 ± 0.01, and 0.26 ± 0.02, respectively). For GOS metabolism, the HT12 and HT13 strains exhibited a strong ability to metabolize than HN9, HT20, and the expression of genes (Balat_0483, Balat_0484, Balat_0475) related GOS metabolism higher than HN9, HT20 strains. The OD_600nm_ vanlues of HT12, HN24, HN7 and HT20 in the medium with XOS as carbon source were 1.05 ± 0.06, 0.87 ± 0.02, 0.42 ± 0.05 and 0.42 ± 0.05 respectively, and the expression levels of XOS-relevant metabolic genes (Balat_0517, Balac_0511, Balac_0514, and Balac_0521) of HT12 and HN24 higher than HN7 and HT20. However, the sample size of the resequencing and reverse transcription validation experimental strains in this study was small, and if further confirmation of intraspecies differences in metabolized oligosaccharides within the *B. animalis* subsp. *lactis* caused by transcriptional rather than the genetic levels is desired, the sample size of the experiments should be increased in subsequent studies in order to overcome the limitations caused by small sample sizes. And generalization of the conclusions to all strains of *B. animalis* subsp. *lactis*.

## 4 Discussion

*B. animalis* subsp. *lactis* are widely distributed in humans and other mammalian guts and are widely supplemented to multiple functional foods. Furthermore, *B. animalis* subsp. *lactis* possesses a saccharolytic lifestyle as well as the ability to utilize multiple oligosaccharides from a variety of host and dietary sources. Multi-omics techniques and the increased number of published *Bifidobacterium* sequences are beneficial for analyzing different strains of genomic multiplicity and the difference in functionality (Andersen et al., 2013; O'Connell Motherway et al., 2013; Nomoto et al., 2017). In this study, we explored the differences in the functional oligosaccharide metabolism of *B. animalis* subsp. *lactis* from three levels: phenotypic characteristics, gene level, and transcription level, and then analyzed the preference and utilization of different oligosaccharides by different strains so as to provide a theoretical reference for the development of suitable synbiotic (“probiotics-prebiotic”) products.

In this study, *B. animalis* subsp. *lactis* were recovery from Hotan, Xinjiang, China and Hainan, China. The results of PCA analysis showed regional variability in the utilization of polysaccharides by *B. animalis* subsp. *lactis* isolated from Hotan and Hainan regions. It could be due to the growth of the *Bifidobacterium* strains may be affected by the environment and eating habits. According to the accumulating evidence, intraspecies differences might occur because of the human gastrointestinal environment (Holscher, 2020). Diet is considered the main environmental contributor to the gastrointestinal microbiota structure and function in humans and animals; therefore, can markedly influence the functionality of indigenous microbiota (Gibson et al., 2004; Hansen et al., 2018). This study resequenced analyzing the strains to explore the mechanism of metabolic oligomerization differences in *B. animalis* subsp. *lactis*.

Studies have shown that about 14% of the genes of Bifidobacteria genome are involved in carbohydrate metabolism (Genomics of the Genus *Bifidobacterium* Reveals Species-Specific Adaptation to the Glycan-Rich Gut Environment). The predominant CAZymes class found in this study were GH43, GT2, GH3, and GT13, which were responsible for the metabolism of multiple plant-derived carbohydrates (Lugli et al., 2019; Liang et al., 2023; Srivastava et al., 2023). In line with this result, our *in vitro* experiments showed that *B. animalis* subsp. *lactis* could assimilate several plant-derived carbohydrates, such as IMO, GOS, and XOS, which may explain why *B. animalis* subsp. *lactis* could efficiently colonize several animal guts. According to *B. animalis* subsp. *lactis* glycobiome, all of the tested strains shared the same CAZymes profile. Nevertheless, our analysis detected strain-specific variation in carbohydrate utilization within 9 *B. animalis* subsp. *lactis*.

However, oligosaccharide utilization capacities of the *B. animalis* subsp. *lactis* strains differed because of the difference in the gene transcription level. In several studies, the *Bifidobacterium* genome functional annotation results revealed that most of the functions encoded by genes were related to carbohydrate metabolism (Milani et al., 2016). The oligosaccharide metabolism experiment combined with the PCR validation of functional genes indicated that the experimental strains could use 5–6 types of oligosaccharides and have genes encoding key enzymes involved in functional oligosaccharide metabolism. This finding suggests that the carbohydrate-utilizing ability of *Bifidobacterium* is crucial for its life activities. The study results are similar to those reported by Janer et al. (2004). In this past study, all experimental strains (*B. animalis* subsp. *lactis*) had genes encoding key enzymes involved in inulin metabolism, as determined through PCR amplification of the gene for β-fructosidase. However, almost none of the strains could metabolize inulin. This finding suggests that although strains possess key enzyme genes for metabolizing functional oligosaccharides, this finding does not confirm that they can surely metabolize relevant oligosaccharides, which could be because the associated genes are not expressed or less expressed. Global gene expression profiles were obtained for 9 *B. animalis* subsp. *lactis*, growing on 4 potential prebiotic oligosaccharides (FOS, GOS, XOS and IMO). RT-qPCR results unveiled that differentiation of gene expression regulation influences metabolic capabilities. HN9, HN10 and HN16 upregulated the expression of glycosyltransferase (Balat_0888) and β-fructofuranosidase (Balat_1241) which are allows to uptake FOS. HN24 and HN5 were observed to utilize IMO via upregulated ABC transporter (Balac_1599), isoamylase (Balat_0977), GH13_311, 6-α-Glucosidase (Balac_1593), 4-α-glucanotransferase (Balat_0373), and glucan phosphorylase (Balat_0076). Transporter differentially up regulated on XOS was also found in HT24, HN7, HT12 and HT20. Glycosidase genes exhibited consistency in terms of phenotype of functional oligosaccharide metabolism at the transcriptional level.

Similarly, the 16th International Symposium on Probiotics and Health reported that probiotics *B. animalis* subsp. *lactis* Probio-M8 and V9 shared similar carbohydrate metabolism-related genes, albeit their phenotypes of carbohydrate metabolism varied remarkably. As noted in the aforementioned report, our study results suggest that all experimental strains (*B. animalis* subsp. *lactis*) had similar genes related to the key enzymes involved in functional oligosaccharide metabolism, but their phenotypes of functional oligosaccharide metabolism were different. The difference in the phenotypes of metabolism may be caused by the difference in the gene transcription level and the protein translation level of relevant key enzymes. Differences were noted in the gene transcription of *Saccharomyces cerevisiae* in the presence of different long-chain fatty acids (Han Li et al., 2019). Moreover, the addition of glycerol effectively increased the transcription level of the lactone ring synthesis-related gene in *Streptomyces ambofaciens*. The relative transcript level of related genes unveiled that the increase in the oxygen supply level effectively promoted the relative transcription level of the forosamine synthesis-related gene. Thus, it was confirmed that the external growth environment can affect gene expression.

## 5 Conclusion

In this study, *B. animalis* subsp. *lactis* strains were isolated from human fecal samples collected from participants from across Hotan and Hainan. Comparison and analyses of their abilities were performed to utilize 8 types of functional oligosaccharides as well as their differences at the gene and transcription levels. The results indicated that, on one hand, the selected strains from different areas exhibited differences in metabolizing functional oligosaccharides. On the other hand, the ability of the strains to utilize the oligosaccharides was consistent with the transcript levels of the related glycosidase genes. This study provided a case for understanding functional diversity due to small gene expression changes within the *B. animalis* subsp. *lactis*. And the synbiotic formulations could be optimized through uncovered gene expression impacts phenotypic variations in *B. animalis* subsp. *lactis*. Subsequent studies could design targeted interventions through understanding the regulatory pathways involved in gene expression, thus developing more effective synbiotic products. This study may provide new ideas for the development of targeted bifidogenic functional oligosaccharides, for specific probiotic strains.

## Data availability statement

The original contributions presented in the study are included in the article/Supplementary material, further inquiries can be directed to the corresponding authors.

## Author contributions

ZL: Formal analysis, Writing – original draft, Writing – review & editing, Validation, Visualization. XZh: Formal analysis, Writing – original draft, Writing – review & editing, Data curation, Software. MX: Data curation, Methodology, Writing – original draft. JK: Writing – review & editing, Investigation. XZu: Investigation, Writing – review & editing. YW: Writing – review & editing, Data curation. CW: Writing – review & editing, Validation. YT: Writing – review & editing, Formal analysis. YN: Writing – review & editing, Resources. YZ: Resources, Writing – review & editing, Conceptualization, Funding acquisition, Project administration.

## Footnotes

^1^ http://tree.bio.ed.ac.uk/software/figtree/
